# Elevated CO_2_ Concentrations Impact the Semiochemistry of Aphid Honeydew without Having a Cascade Effect on an Aphid Predator

**DOI:** 10.3390/insects9020047

**Published:** 2018-04-20

**Authors:** Antoine Boullis, Solène Blanchard, Frédéric Francis, François Verheggen

**Affiliations:** Laboratory of Functional and Evolutionary Entomology, Gembloux Agro-Bio Tech, University of Liege (ULg), Passage des déportés, 2-5030 Gembloux, Belgium; aboullis@pasteur-guadeloupe.fr (A.B.); solene.blanchard@uliege.be (S.B.); frederic.francis@uliege.be (F.F.)

**Keywords:** climate change, carbon dioxide, *Acyrthosiphon pisum*, *Episyrphus balteatus*, searching behavior, honeydew production, volatile organic compounds

## Abstract

Honeydew is considered a cornerstone of the interactions between aphids and their natural enemies. Bacteria activity occurring in aphid honeydew typically results in the release of volatile organic compounds (VOCs) that are used by the natural enemies of aphids to locate their prey. Because atmospheric carbon dioxide (CO_2_) concentration directly impacts the physiology of plants, we raise the hypothesis that elevated CO_2_ concentrations impact the quantity of honeydew produced by aphids, as well as the diversity and quantity of honeydew VOCs, leading to cascade effects on the foraging behavior of aphids’ natural enemies. Using solid-phase microextraction, we analyzed the VOCs emitted by honeydew from pea aphids (*Acyrthosiphon pisum* Harris) reared under 450 ± 50 ppm of CO_2_ (aCO_2_) or 800 ± 50 ppm CO_2_ (eCO_2_). While the total amount of honeydew excreted was only slightly reduced by eCO_2_ concentrations, we detected qualitative and quantitative differences in the semiochemistry of aphid honeydew between CO_2_ conditions. Three VOCs were not found in the honeydew of eCO_2_ aphids: 3-methyl-2-buten-1-ol, 2-methyl-1-butanol, and isobutanol. However, no difference was observed in the searching and oviposition behaviors of hoverfly (*Episyrphus balteatus* (De Geer)) females exposed to plants covered with honeydew originating from the different CO_2_ conditions. The present work showed the effect of a particular aspect of atmospheric changes, and should be extended to other abiotic parameters, such as temperature.

## 1. Introduction

Aphid honeydew is not just a waste product of sap-feeder insects [1], it is also a key element in the interactions between aphids and other organisms, such as aphid predators (e.g., [2]), parasitoids (e.g., [3]), and mutualistic organisms (e.g., [4]). This aqueous mixture is mainly made of sugars (up to 90% dry weight), but it also contains minerals and amino acids [5]. Honeydew composition is affected by the species or cultivar of host species [6]. The amino acid composition qualitatively reflects phloem sap content [7]. Most of the honeydew sugars are directly transferred from the phloem, but some are synthesized by aphids or through bacterial enzymatic activity [8].

Due to its rich composition in carbohydrates and nitrogen-based molecules, honeydew also constitutes an optimal growth medium for microorganisms. Bacteria from the genus *Staphylococcus* were identified from honeydew and contribute to the production of volatile organic compounds (VOCs) in two aphid species [9,10]. Some of these compounds act as kairomones for aphid predators or synomones for mutualistic partners [10,11]. These volatile non-cyclic compounds are by-products originating from the degradation of amino acids and sugars in honeydew [10]. Carbon dioxide (CO_2_) atmospheric concentration impacts plant growth and physiology [12]. It also impacts the ability of *Staphylococcus* bacteria to exploit the resources in their environment [13]. However, how predators respond toward elevated CO_2_ concentration is difficult to predict, due to multiple interactions occurring in this multitropic system [14]. Thus, we hypothesize that elevated CO_2_ concentrations impact the emission of VOCs from honeydew, leading to a cascade effect on the ability of aphids’ natural enemies to locate their prey. In this study, we reared two groups of pea aphids (*Acyrthosiphon pisum* Harris) under two contrasting CO_2_ concentrations. We measured (1) the production of honeydew; (2) the VOC profile released by the honeydew; and (3) the resulting impact on the searching and oviposition behaviors of the aphid predator *Episyrphus balteatus* (De Geer). Our results are expected to show how atmospheric changes influence the interactions between aphids and their predators.

## 2. Materials and Methods

### 2.1. Plants and Insects

Two *A. pisum* populations originating from a single individual (clone L1-22) were reared on the broad bean *Vicia faba* L. (var. “Major”) under two different CO_2_ concentrations for at least 100 generations (about two years of parthenogenetic development). These populations were reared in 12 climatic chambers previously described in [15]. In summary, half of these chambers were maintained at an ambient concentration of 450 ± 50 ppm (termed aCO_2_) and the other half were maintained at a CO_2_ concentration enriched by the addition of 350 ppm (termed eCO_2_) by using a CO_2_ gas tank (>99% purity; AirLiquide, Paris, France). Temperature (23 ± 1 °C), relative humidity (60 ± 10%), photoperiod (16/8 h light/dark), light intensity (35 µmol m^−2^ s^−1^ at canopy level), and the watering of plants was identical among all chambers. Host plants were grown in plastic trays (30 × 20 × 6 cm) containing a perlite:vermiculite substrate (proportion 1:1) and were watered abundantly. Plants were maintained under their respective CO_2_ concentrations from the seedling stage. After one week of growing, aphids were transferred from old trays to new ones to ensure their proper development. The growing stages were identical under both CO_2_ concentrations (corresponding to the phenological stage 10 on the BBCH scale). Planting of seeds and aphid transfer were renewed each week. To minimize the potential effect of heterogeneity among chambers, plants and aphids were moved from one chamber to another once a week.

A population of *E. balteatus* was maintained under laboratory conditions in netted flight cages (75 × 60 × 90 cm) and was fed with fresh pollen, honey, and sugar. The food source was renewed every 10 days. To induce oviposition, broad bean plants infested with pea aphids *A. pisum* were placed inside the flight cage for 24 h. After hatching, hoverfly larvae were fed ad libitum with pea aphids until pupation. Adults were then placed in flight cages, sorted by emergence date.

### 2.2. Honeydew Collection

Plants infested with *A. pisum* grown under both CO_2_ conditions were placed above sterilized plastic foils and the dripping droplets were collected with capillary tubes (100 mm length × 0.95 mm I.D.; Minicaps^®^ 10 µL for honeydew quantification; Hirschmann^®^ Laborgerate, Eberstadt, Germany). The collected honeydew was stored in 200 µL glass inserts at room conditions. Honeydew from the two different CO_2_ treatments were collected and stored under the same laboratory conditions (T: 21 ± 1 °C; RH: 60 ± 10%; [CO_2_]: 450 ± 50 ppm).

### 2.3. Quantification of Honeydew

We quantified the honeydew being excreted by colonies of adult *A. pisum* originating from different CO_2_ concentrations. To do so, we started by standardizing the age of the aphids. *V. faba* plants var. “Major” were grown under aCO_2_ and eCO_2_ conditions. After nine days (corresponding to phenological stage 10 on the BBCH scale), plants were isolated in pairs in plastic pots (7 × 7 × 8 cm) and infested with 20 apterous adult aphids from the mass rearing of the respective CO_2_ conditions. Two days after infestation, adult aphids were removed, and their offspring was used six days afterwards in this experiment. The honeydew was collected on each infested plant for five hours (see Section 2.2). The volume collected during this period was measured based on the number of capillaries filled with honeydew. We also compared the volume of honeydew droplets between both aphid populations. To do so, we counted the number of droplets that were necessary to fill a 10 µL capillary completely. To avoid any bias in observations, the experimenter was not informed about the CO_2_ conditions that the aphid colonies belonged to. After the experiment, the number of aphids on each plant was counted and weighed using an analytical balance (Kern ABT 120-5DM; readout: 0.01 mg; Kern & Sohn GmbH, Balingen, Germany). Nine and 11 replicates were performed for the aCO_2_ and eCO_2_ conditions, respectively.

### 2.4. Identification and Estimated Quantity of Honeydew Semiochemicals

The VOC emissions of the honeydew were compared among aphids originating from the two CO_2_ conditions. VOC collection was performed by solid-phase microextraction (SPME) on 20 µL crude honeydew collected from a glass insert, by using a 10-mm fiber with a 50/30 µm carboxen–divinylbenzene–polydimethylsiloxane coating (DVB/CAR/PDMS; Supelco, Bellefonte, PA, USA). Six distinct fibers were used to collect VOCs from (i) an empty insert (control); (ii) honeydew from aCO_2_ aphids; and (iii) honeydew from eCO_2_ aphids (*n* = 6 for each condition). The experiment took place over three consecutive days. Volatile collection was performed under laboratory conditions (T: 22 ± 1 °C; RH: 60 ± 10%; [CO_2_]: 450 ± 50 ppm). Before sampling, each SPME fiber was conditioned in a gas chromatography injector at 250 °C for 55 min.

We followed a previously published methodology to separate and identify honeydew VOCs [10]. We used an Agilent Technologies© (Santa Clara, CA, USA) 6890 gas chromatograph coupled to an Agilent 5973 mass spectrometer (GC–MS). The capillary column was an Agilent HP-5MS (5% phenyl methyl; 30 m length; 0.25 mm I.D., 0.25 μm film thickness), and helium was used as the carrier gas (constant flow of 1 mL min^-1^). The injector was set at 230 °C and was on splitless mode. The oven temperature was first held at 40 °C for 2 min, then was raised at 5 °C/min to 150 °C, at 10 °C/min to 210 °C, at 120 °C/min to 280 °C, and finally was held for 1 min at 280 °C. Mass spectra were taken at 70 eV, with a mass range extending from *m*/*z* 35 to 350 amu. Mass spectra were interpreted for identification, before being confirmed by the injection of synthetic standards.

To estimate the quantity of the compounds, three concentrations of standard blend solutions of the different identified VOCs diluted in methanol (>97% purity; VWR International, Leuven, Belgium) were injected under the same GC–MS conditions. Each concentration was injected three to five times, and a calibration curve was established by using the method of least square fit analysis (see supplementary data Table S1 and Figure S1). The areas of peaks obtained during odor sampling were compared to the calibration curve to estimate the quantity.

### 2.5. Behavioral Experiment

Because the results of the previous experiment obtained different semiochemistry in the honeydew of aphids reared under aCO_2_ and eCO_2_ concentrations, we tested the hypothesis of a cascade effect on the searching and oviposition behaviors of an aphid predator. Young plants (i.e., phenological stage 10 on the BBCH scale) grown either under aCO_2_ or eCO_2_ conditions were isolated in plastic containers (50 cm^3^) filled with perlite:vermiculite substrate and 30 mL of water. The plants were covered with 35 µL fresh honeydew that had been collected from the corresponding CO_2_ treatment (see Section 2.2). This amount was previously shown to induce oviposition in hoverflies [16,17]. According to our quantification assays, this volume represents a quantity of honeydew excreted by 50 adult aphids over 24 h. After honeydew deposition, plants were left over for 30 min to let the honeydew dry. The behavioral experiment started with the introduction of a single mated and gravid hoverfly female in a flight cage (25 × 25 × 65 cm; Bugdorm, Megaview Science, Taiwan) in the presence of two plants, one from each CO_2_ condition. One hour after the beginning of the experiment, behavioral observations were made using the software The Observer XT (Noldus Technology©, Wageningen, The Netherlands) for 30 min. During observations, the duration of the following behaviors were recorded: (i) random flight and landing on the cage; (ii) stationary-oriented flight near a plant; (iii) walking on a plant; and (iv) oviposition (the hoverfly extended its abdomen and touched the plant stem or leaf with its ovipositor). Here again, the experimenter was considered naïve, with no knowledge of the CO_2_ condition that the plant and honeydew were obtained from. The number of eggs laid on each plant was counted for four hours after introducing the female. The experiments were conducted under laboratory conditions (T: 21 ± 1 °C; RH: 60 ± 10%; [CO_2_]: 450 ± 50 ppm). Hoverfly females were 17 to 24 days old and had not previously been exposed to aphid products. The behavior of 20 females was recorded.

### 2.6. Statistical Analyses

All statistical tests were conducted using software R version 3.0.1 [18]. All the values presented below are means ± standard error (SE). Comparison of honeydew production by aphid colonies between both CO_2_ treatments was assessed using a Student *t*-test. The same test was applied to compare droplet production per aphid and droplet volume between CO_2_ conditions. Before using these parametric tests, the normality of data and homoscedasticity were checked using the Shapiro–Wilk and Bartlett tests, respectively (*p* > 0.05). A two-way analysis of variance (ANOVA) was applied to compare the VOC profiles from both blends of honeydew. The quantity of VOCs was considered as the response variable, while CO_2_ concentrations and compounds were considered as explanatory variables. The test was only applied on compounds that were found in both CO_2_ treatments. A Tukey’s honesty significant difference (HSD) test was then applied to compare the means individually. Concerning behavioral assays, a Student *t*-test was applied to compare the mean durations of each observed behavioral trait. Finally, clutch size on each plant was compared using a paired Student *t*-test. Only the females that oviposited and exhibited searching behavior during the test were used in the statistical analyses.

## 3. Results

### 3.1. Quantification of Honeydew

Aphid colonies consisted of of 167 ± 28 and 190 ± 16 individuals for aCO_2_ and eCO_2_ conditions, respectively. An aphid had a mean weight of 2.09 ± 0.11 and 2.13 ± 0.06 mg, respectively (Student *t*-test: *t* = −0.332, *p* = 0.744). The amount of honeydew produced per aphid did not differ between aCO_2_ and eCO_2_ individuals, with 0.95 ± 0.07 and 0.75 ± 0.07 µL aphid d^−1^, respectively (Student *t*-test: *t* = 1.968, *p* = 0.065). However, when considering an equal aphid weight, colonies reared under aCO_2_ concentrations produced significantly more honeydew than those reared under eCO_2_ conditions, with 45.26 ± 3.40 µL 100 mg aphid d^−1^ versus 34.86 ± 2.98 µL 100 mg aphid d^−1^, respectively (Student *t*-test: *t* = 2.305, *p* = 0.034). The number of droplets produced by aphids reared under aCO_2_ conditions (5.81 ± 0.69 droplets over 24 h) did not differ to those under eCO_2_ conditions (4.92 ± 0.62 droplets over 24 h) (Student *t*-test: *t* = 0.963, *p* = 0.349). The volume of the droplets did not vary significantly between the two CO_2_ conditions (Student *t*-test: *t* = 1.382, *p* = 0.185). Overall, 50 droplets produced by aCO_2_ aphids represented 7.20 ± 0.45 µL, while 50 droplets produced by eCO_2_ aphids represented 6.03 ± 0.71 µL. 

### 3.2. Honeydew Semiochemical Analysis

**Figure 1:**
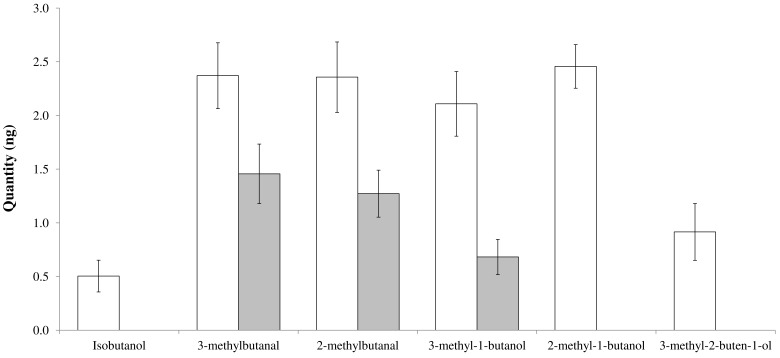
Amount (ng ± standard error (SE)) of the different volatile organic compounds (VOCs) emitted by the honeydew of *A. pisum* reared under aCO_2_ (**white bars**) and eCO_2_ (**grey bars**) conditions.

Ten volatile molecules were collected and identified from the honeydew of aCO_2_ aphids (Table 1). Acetaldehyde, 2-thiapropane, ethanol, and 2-propanone were detected in all replicates, but not quantified (the first two were under the limit of quantification, the last two co-eluted with the solvent in the quantification purpose). The quantities of other detected molecules were estimated by using different linear correlations obtained with the calibration curves (Supplementary Table S1 and Figure S1). Curves were developed by using the areas of each compound*_i_* (x*_i_*) and the quantity of compound*_i_* injected (y*_i_*). Acetaldehyde was not detected from eCO_2_ aphids. Six compounds where quantified from aCO_2_ aphids (Figure 1); namely, isobutanol, 3-methylbutanal, 2-methylbutanal, 3-methyl-1-butanol, 2-methyl-1-butanol, and 3-methyl-2-buten-1-ol. Isobutanol, 2-methyl-1-butanol, and 3-methyl-2-buten-1-ol were not observed in eCO_2_ aphids. More 3-methyl-1-butanol was found in aCO_2_ aphids (Tukey’s HSD: *p* = 0.009).

### 3.3. Behavioral Experiments

**Figure 2:**
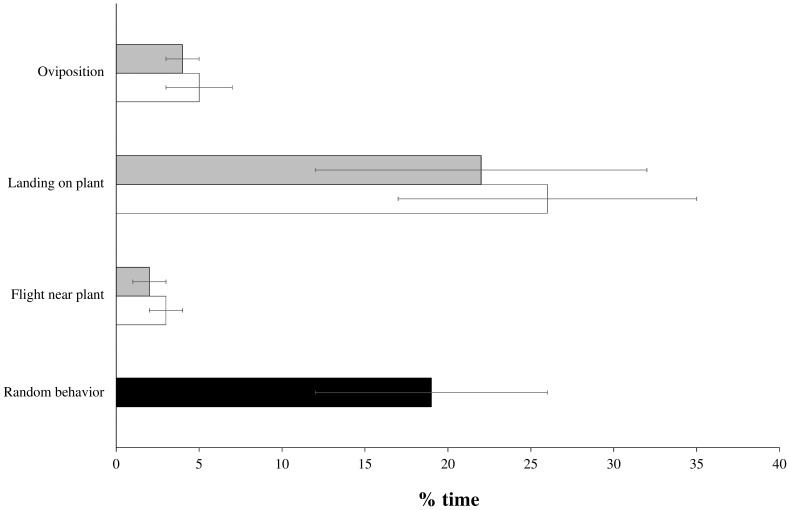
Percentage of time (±SE) that hoverfly females expressed different behaviors on plants smeared with crude honeydew from aCO_2_ (**white bars**) and eCO_2_ (**grey bars**) conditions in a dual-choice assay. The random choice category (**black bar**) was shared for both CO_2_ conditions.

**Figure 3:**
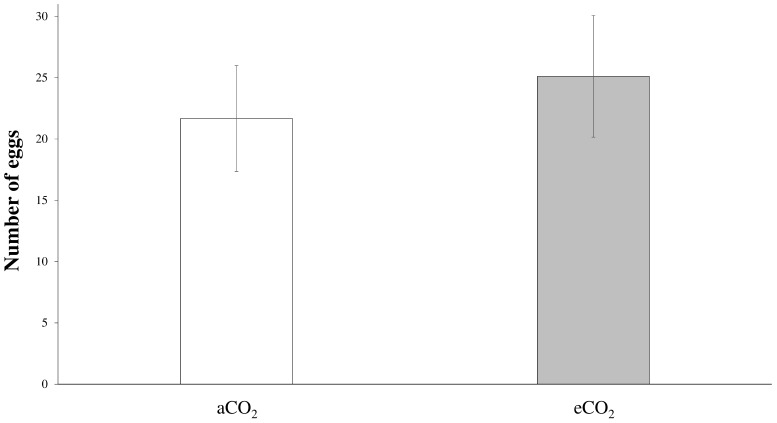
Mean (±SE) number of eggs laid by the hoverfly females on plants smeared with crude honeydew from aCO_2_ (**white bars**) and eCO_2_ (**grey bars**) conditions.

Nine of the 20 tested females exhibited searching and oviposition behaviors. Out of these responding females, no preference was detected between plants covered with honeydew originating from aCO_2_ or eCO_2_. The duration of the flight near each plant was similar (Student *t*-test: *t* = 2.020, *p* = 0.078), as was the time spent on each plant (Student *t*-test: *t* = 0.221, *p* = 0.831) and the time spent laying eggs (Student *t*-test: *t* = 0.439, *p* = 0.672) (Figure 2). These hoverfly females laid 22 ± 4 eggs on plants covered with aCO_2_ honeydew and 25 ± 5 eggs on plants covered with eCO_2_ honeydew (Student *t*-test: *t* = −1.557, *p* = 0.158) (Figure 3).

## 4. Discussion

Based on our results, we suggest that the honeydew volatilome is impacted by atmospheric CO_2_ concentrations. Specifically, elevated CO_2_ concentrations lead to a lower diversity and quantity of honeydew VOCs. Acetaldehyde, isobutanol, 2-methyl-1-butanol and 3-methyl-2-buten-1-ol were only found from aCO_2_ honeydew. Also, 3-methyl-1-butanol was more abundant in aCO_2_ honeydew. To date, the volatile compounds released by aphid honeydew have been identified in three aphid species, including *A. pisum* [2,9,10]. Four of the molecules identified in the present study were also found by Leroy et al. [9]; namely 2-propanone, 3-methylbutanal, 2-methylbutanal, and 3-methyl-1-butanol. We identified additional VOCs, including 2-methyl-1-butanol, which is also released by the honeydew of the two other aphid species, *Megoura viciae* Buckton [2] and *Aphis fabae* Scopoli [10]. These volatile molecules are the results of direct modifications of amino acid-derived starter units, performed by bacteria [19]. We found additional molecules that were not previously detected in aphid honeydew, including acetaldehyde, 2-thiapropane, isobutanol, and 3-methyl-2-buten-1-ol. The observed differences in VOC emissions might be related to different rearing conditions, aphid strains and associated microbial flora.

Whether the honeydew bacteria community (diversity and abundance) is impacted by CO_2_ concentration needs investigation. Amino acids and sugars present in honeydew are considered as precursors for bacterial metabolism [10]. Phloem sap composition might be modified under increased CO_2_ concentrations, due to the primary and secondary metabolism of host plants being modified [20]. However, the impact of CO_2_ on phloem characteristics remains unclear, with some authors finding that free amino acid content decreased [21,22,23], showed no significant modification [24,25], or increased [26]. Because honeydew composition reflects phloem content, its nutrient composition might also be affected by CO_2_ concentrations.

The volatile cues released by aphid honeydew have been shown to guide aphid predators toward their prey, and to induce oviposition [27]. In our behavioral assay, gravid females of *E. balteatus* used both aphid-infested plants (aCO_2_ and eCO_2_) as appropriate oviposition sites. The slight qualitative and quantitative differences in VOCs highlighted previously were not sufficient to allow ovipositing females to discriminate the two plants.

We also hypothesized that the amount of excreted honeydew is impacted by CO_2_ concentrations. Indeed, due to possible changes in phloem sap, aphids might adapt their feeding behavior, and thus the ingestion of sap, to reach their nutritional threshold. We found that aphid colonies (in terms of weight) reared under enriched CO_2_ concentrations reduce the quantity of honeydew emitted, but do not influence the number and size of the droplets excreted by aphids. Contrasting results were previously obtained, suggesting that aphids reared under elevated CO_2_ concentrations produce less [22] or more honeydew [28]. However, the species used and the physiological status of the host plant might influence the quality of excreted honeydew.

## 5. Conclusions

A higher atmospheric CO_2_ concentration modifies the semiochemistry of aphid honeydew. However, we found no cascade effect on the prey-searching behavior of the aphid predator *E. balteatus*. Because CO_2_ is just one isolated component of climate change, we suggest that additional studies should strengthen our knowledge of the impact of combined abiotic parameters on insect herbivory. Taking into account multitrophic interactions is also important for evaluating the impact on these functional aspects.

## Figures and Tables

**Table 1:** Volatile organic compounds emitted from the honeydew of aphids reared under aCO_2_ and elevated CO_2_ concentrations. Values in bold indicate significant differences between CO_2_ conditions.

Compound	CAS #	Retention Time (min)	aCO_2_	eCO_2_	*P*
Acetaldehyde	75-07-0	3.406	D	-	-
Ethanol	64-17-5	3.592	D	D	-
2-propanone	407-25-0	3.756	D	D	-
2-thiapropane	75-18-3	3.939	D	D	-
Isobutanol	78-83-1	4.958	**0.504 ± 0.147**	-	-
3-methylbutanal	590-86-3	5.391	2.371 ± 0.306	1.456 ± 0.277	0.197
2-methylbutanal	96-17-3	5.557	2.357 ± 0.328	1.272 ± 0.219	0.081
3-methyl-1-butanol	123-51-3	7.038	**2.108 ± 0.301**	0.682 ± 0.164	0.009
2-methyl-1-butanol	137-32-6	7.077	**2.458 ± 0.203**	-	-
3-methyl-2-buten-1-ol	556-82-1	8.201	**0.916 ± 0.264**	-	-

## Supplementary Materials

The following are available online at http://www.mdpi.com/2075-4450/9/2/47/s1. Figure S1: Calibration curves of different volatile organic compounds from aphid honeydew obtained by the least squares fit analysis method. The quantity of four compounds was estimated: isobutanol (A), 3-methyl-2-buten-1-ol (B), 2-methyl-1-butanol (C), and 3-methyl-1-butanol (D). Table S1: Characteristics of the calibration curves and synthetic standards of the different volatile organic compounds from aphid honeydew.
