# Supplementary material for: Elevated CO2 Concentrations Impact the Semiochemistry of Aphid Honeydew without Having a Cascade Effect on an Aphid Predator
Source: Insects. 2018 Apr 20;9(2):47. doi: 10.3390/insects9020047 (PMC6023513; doi:10.3390/insects9020047)
Supplement: Supplementary file 1 [file insects-09-00047-s001.pdf]

Supplementary material

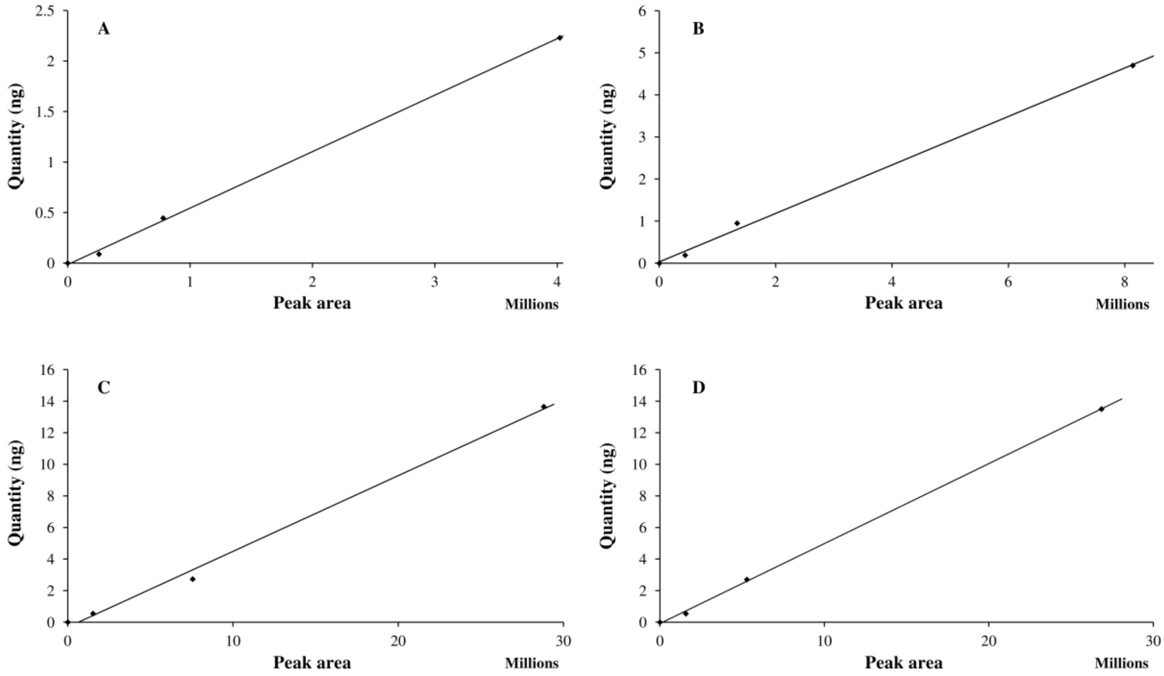

Figure S1. Calibration curves of different volatile organic compounds from aphid honeydew obtained by the least squares fit analysis method. The quantity of four compounds was estimated: Isobutanol (A), 3-methyl-2-buten-1-ol (B), 2-methyl-1-butanol (C), and 3-methyl-1-butanol (D).

Table S1. Characteristics of the calibration curves and synthetic standards of the different volatile organic compounds from aphid honeydew.

**Compound**	CAS #	Purity (%)	Linear correlation (y =)	R^2
Isobutanol	78-83-1	> 99	$6.10^{-07}x - 0.0152$	0.9993
3-methyl-1-butanol[a]	123-51-3	> 99	$5.10^{-07}x - 0.0826$	0.9996
2-methyl-1-butanol[a]	137-32-6	> 99	$5.10^{-07}x - 0.3117$	0.9961
3-methyl-2-buten-1-ol	556-82-1	99	$6.10^{-07}x + 0.0315$	0.9963

[a] Quantity of 3-metylbutanal and 2-methylbutanal were estimated with the linear correlations of their corresponding alcohols
